# Mr. Smallwood's Complaint

**Published:** 1918-02-09

**Authors:** Mabel Meysey-Thompson

**Affiliations:** 74 Oxford Terrace, Hyde Park, W.


### Mr. Smallwood's Complaint.

*To the Editor of* The Hospital.

Sir,—As Mr. Smallwood, M.P., has given what purports to be his experience of Hospital No. 20 in France, perhaps I may be permitted to give mine.

My son, Captain Meysey-Thompson, was grievously wounded in September 1917, and in consequence I was telegraphed for and went to see him. Nothing could exceed the consideration and forethought shown by the medical authorities, and particularly those connected with this hospital, in the arrangements made for my journey and reception. The matron of the above hospital is well known for her aptitude and the exceptional kindness she has shown both to the patients and their relatives. My son has now been four months in this hospital, and he cannot speak too highly of their attention. He writes to me to say that the greatest indignation is felt amongst the patients at the allegations made by Mr. Smallwood, whose statements do not tally with the recollections of those who are in the hospital. My son is a barrister of over thirty years of age and accustomed to weigh evidence, and he is most careful in his statements.

I write this letter because I think it right that the public should know that the reputation which this hospital generally enjoys is widely different from the character which Mr. Smallwood chose to give it.—Yours faithfully, Mabel Meysey-Thompson.

74 Oxford Terrace, Hyde Park, W.,
February 1, 1918.

